# A Copper-Based Biosensor for Dual-Mode Glucose Detection

**DOI:** 10.3389/fchem.2022.861353

**Published:** 2022-04-04

**Authors:** Kai Li, Xiaoyu Xu, Wanshan Liu, Shouzhi Yang, Lin Huang, Shuai Tang, Ziyue Zhang, Yuning Wang, Fangmin Chen, Kun Qian

**Affiliations:** ^1^ Department of Urology, Tianjin Third Central Hospital Affiliated to Nankai University, Tianjin, China; ^2^ State Key Laboratory for Oncogenes and Related Genes, School of Biomedical Engineering, Institute of Medical Robotics and Med-X Research Institute, Shanghai Jiao Tong University, Shanghai, China; ^3^ Division of Cardiology, Renji Hospital, School of Medicine, Shanghai Jiao Tong University, Shanghai, China; ^4^ Department of Clinical Laboratory Medicine, Shanghai Chest Hospital, Shanghai Jiao Tong University, Shanghai, China

**Keywords:** Cu_2_O nanoparticle, colorimetric, mass spectrometry, glucose, biosensor

## Abstract

Glucose is a source of energy for daily activities of the human body and is regarded as a clinical biomarker, due to the abnormal glucose level in the blood leading to many endocrine metabolic diseases. Thus, it is indispensable to develop simple, accurate, and sensitive methods for glucose detection. However, the current methods mainly depend on natural enzymes, which are unstable, hard to prepare, and expensive, limiting the extensive applications in clinics. Herein, we propose a dual-mode Cu_2_O nanoparticles (NPs) based biosensor for glucose analysis based on colorimetric assay and laser desorption/ionization mass spectrometry (LDI MS). Cu_2_O NPs exhibited excellent peroxidase-like activity and served as a matrix for LDI MS analysis, achieving visual and accurate quantitative analysis of glucose in serum. Our proposed method possesses promising application values in clinical disease diagnostics and monitoring.

## Introduction

Glucose plays important role in the human body, providing energy for metabolism and normal operation of various organs (Grochowska et al., 2017). As is reported, disorders of glucose metabolism cause diabetes (Zheng et al., 2018), hyperglycemia (Beaudry et al., 2013), or other diseases (Sardarinia et al., 2016). Glucose is regarded as a biomarker in the clinic, the concentration of which reflects the abnormal behavior of the body. For example, the glucose level in cancer cells is higher than that in healthy cells (Wu et al., 2019). Thus, it is indispensable to develop simple, accurate, and sensitive methods for glucose detection.

At present, the usually used methods for glucose detection mainly depend on enzymatic reactions (Aksorn and Teepoo, 2020; Kim et al., 2020). In brief, glucose could be transferred to hydrogen peroxide (H_2_O_2_) and gluconic acid with the help of glucose oxidase (GOD). The generated H_2_O_2_ catalyzes the substrates to colored products in the presence of peroxidase, which could be analyzed by electrochemical sensors or optical sensors (Qu et al., 2021). Specifically, colorimetric biosensors with the advantages of simplicity, visualization, and low cost, have been widely used in glucose detection (Liu et al., 2019). However, the natural enzymes are unstable, hard to prepare and expensive limiting the extensively clinical applications (Ambati and Jachak, 2021). Since the nanoenzymes come out (Mei et al., 2020; Huo et al., 2022), various nanomaterials possessing peroxidase-like activity have been successfully utilized to detect glucose (Jia et al., 2016). Remarkably, it is important to fabricate the easily prepared nanomaterials-based peroxidase-like colorimetric biosensors for glucose analysis.

Currently, matrix-assisted laser desorption/ionization mass spectrometry (MALDI MS) has been widely applied in biomolecules analysis (Li et al., 2020; Wang et al., 2021). Compared to colorimetric assay, MALDI MS presents high sensitivity, accuracy, throughput and provides molecular information (Israr et al., 2020). Nevertheless, it is a challenge to quantitatively analyze small molecules due to the background signal and coffee effect of traditional organic matrices (Wu et al., 2018). In recent years, plenty of research has been paid more attention to nanomaterials-assisted LDI MS for metabolic analysis (Cao et al., 2020; Liu et al., 2020; Ding et al., 2022). In the process of LDI MS, the matrix can uniformly distribute among the targets (Dai et al., 2020) and enhance the ionization efficiency, largely improving the MS quantitative performance (Kim et al., 2021).

Herein, we propose a copper-based biosensor for dual-mode glucose analysis based on colorimetric assay and LDI MS (Schem 1). Cu_2_O nanoparticles (NPs) were facilely prepared and testified the peroxidase-like activity, achieving visual detection of glucose combined with GOD. Meanwhile, Cu_2_O NPs were utilized as a matrix for LDI MS analysis of small molecules (e.g. glucose) with good salt tolerance. By adding internal standard, glucose level could be quantitative analysis by Cu_2_O NPs assisted LDI MS. Notably, the dual-mode Cu_2_O NPs based biosensor was applied to detect glucose from serum and a consistent result was obtained, demonstrating that the method could be reliable in glucose analysis for clinical diseases diagnostics and monitoring.

**SCHEME 1:**
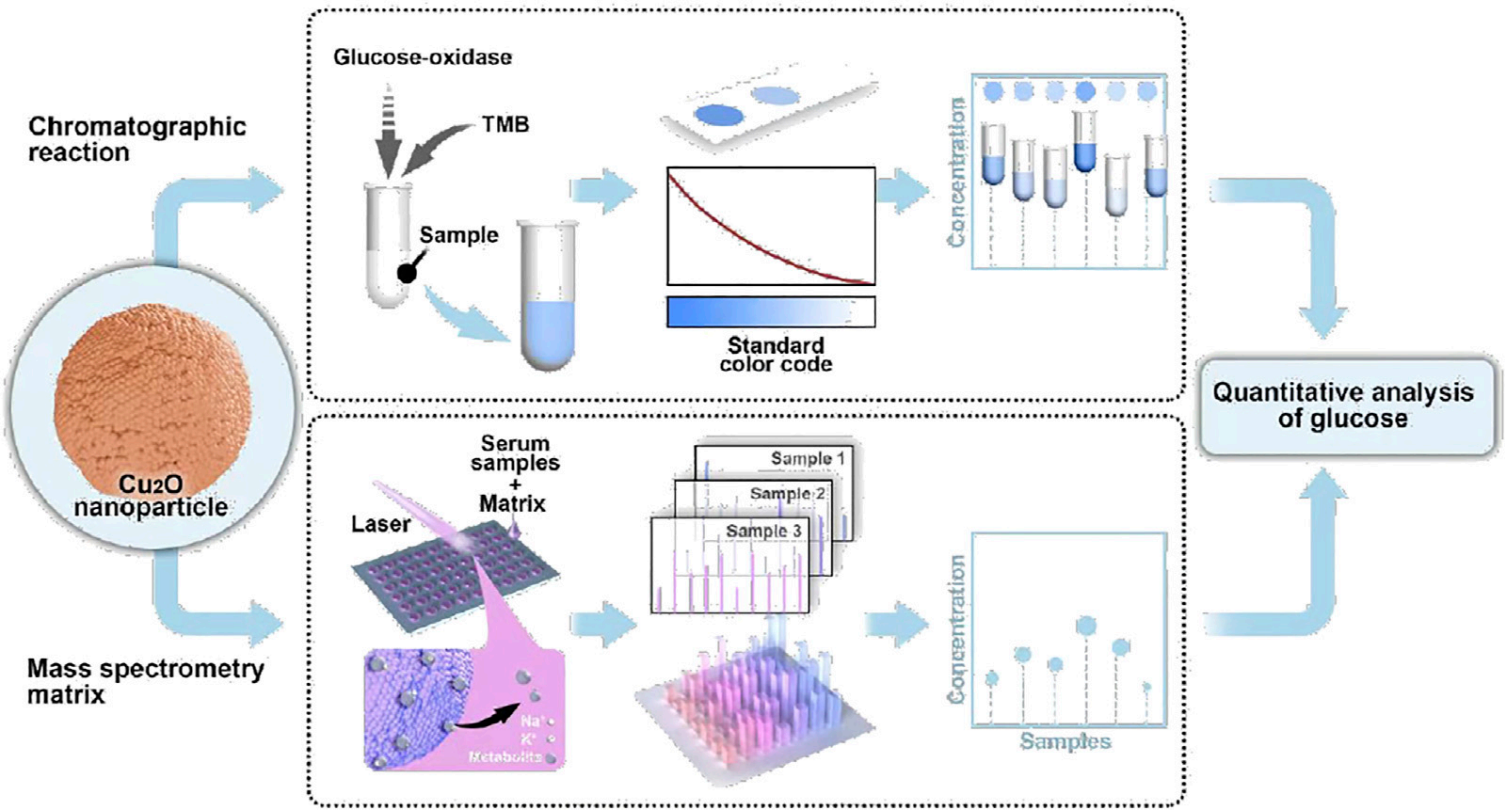
Schematic workflow of the copper-based biosensor for dual-mode glucose detection.

### Experimental Section

#### Chemicals and Materials

CuSO_4_ 5H_2_O (98%), sodium hydroxide (NaOH, 96%), D-(+)-glucose (Glu, 99.5%), ethylene glycol (99%), D-(+)-cellobiose (Cel, 98%), L-arginine (Arg, 98%), d-phenylalanine (98%), anhydrous ethanol (EtOH, 99.7%), sodium acetate (98.5%), choline (Cho, 90%), ascorbic acid (Vc, 99%), d-galactose (Gla, 95%), and dopamine (DA, 98%) were acquired from Inno-chem Co., Ltd. (Beijing, China). Polyvinylpyrrolidone (PVP, MW = 40,000), L-aspartic acid (Asp, 98%), D-(-)-fructose (Fru, 98%), L-alanine (Ala, 99%), 3,3′,5,5′-tetramethylbenzidine (TMB, 99%), and acetic acid (99%) were ordered from Sigma, United States. Dimethylsulfoxide (DMSO, 99.8%) was bought from Aladdin Reagent Co., Ltd. (Shanghai, China). Phosphate-buffered saline (PBS, 10×, pH 7.4, cell-culture grade) and Sodium acetate (98.5%) were ordered from Tokyo Chemical Industry Co., Ltd. (Tokyo, Japan). Glucose oxidase (GOD) was acquired from Shanghai Macklin Biochemical Co., Ltd. (Shanghai, China). All chemicals were used without any further purification unless otherwise stated. Deionized (DI) water (18.2 MΩ cm) was prepared by a Milli-Q water purification system (Millipore, Billerica, MA).

#### Instruments and Characterization

Transmission electron microscopy (TEM), high-resolution transmission electron microscopy (HRTEM), high-angle annular dark-filed (HAADF), and elemental mapping images were collected using a JEOL JEM-2100F instrument by depositing materials on a copper grid with a mesh size of 200. Scanning electron microscopy (SEM) images were recorded on Hitachi S-4800 by dropping the ethanolic material suspensions on aluminum foil. The crystal structure of the wide-angle powder pattern was analyzed through X-ray diffraction (BrukerD8, Germany) with Cu Kα radiation (*λ* = 0.154 nm). The materials absorption spectra were obtained on an Ultraviolet-visible (UV-Vis) (AuCy UV1900, China) spectrophotometer and MD SpectraMax i3x using water suspension at room temperature (RT, 25°C). The LDI MS analysis was performed on Matrix-Assisted Laser Desorption/ionization Time of Flight Mass Spectrometry (MALDI-TOF MS, Bruker Autoflex Speed, Germany) with the Nd: YAG laser (2 kHz, 355 nm) and smart beam system. The acquisitions were conducted in positive reflector ion mode with the repetition rate of 1 kHz and an acceleration voltage of delayed extraction set as 20 kV. Optimized delay time for this experiment to 250 ns and laser shots of 2000 per detection was applied throughout LDI MS analysis. For the calibration of each spot, dropped mixed small molecules for the accurate measurement of mass spectrometry (<20 ppm). Each sample detection performed five independent experiments and all spectra results were used for analysis directly without any smoothing procedures.

#### Preparation of Biofluid

Blood samples were donated by healthy controls and patients in Tianjin Third Central Hospital. The serum samples were prepared from blood according to the previous report (Huang L. et al., 2019). 3 ml of blood was drawn to BD Vacutainer SST tubes (Becton, Dickinson and Co., United States) and centrifuged at 3,000 rpm for 15 min to collect aliquots of the supernatant as serum. All samples were harvested in tubes and stored at −80°C for use. All the investigation protocols in this study were approved by the institutional ethics committees of Tianjin Third Central Hospital and School of Biomedical Engineering, Shanghai Jiao Tong University (SJTU). Informed consent from healthy controls and patients had been obtained since the project started.

#### Synthesis of Cu_2_O Nanoparticles

The Cu_2_O NPs were prepared according to the improved glucose reduction method (Sheng et al., 2020). Briefly, 0.2496 g of CuSO_4_.5H_2_O and 0.04 g of PVP were dispersed in 50 ml of ethylene glycol for 30 min under ultrasonic vibration. Then, 25 ml of NaOH (0.1 M) were added and stirred at RT for 10 min. After that, 25 ml of glucose (1.3 M) were added with slowly stirring for 15 min and the obtained mixture was heated at 80°C for 1 h in a water bath. After the resulting solution cooled to RT naturally, the orange sediment was collected and washed several times thoroughly with DI water and EtOH by centrifugation. Finally, the Cu_2_O NPs were obtained by drying for 5 h in a vacuum oven at 55°C.

#### Peroxidase Activity of the Cu_2_O NPs

The Cu_2_O NPs have the property of peroxidase, which was verified by the oxidation of TMB in the presence of H_2_O_2_. In a typical procedure, 100 µl of TMB (20.8 mM, dissolved in DMSO), 100 µl of H_2_O_2_ (5 mM) and 5 µl of Cu_2_O NPs (7 mM) were added in 400 µl of HAc-NaAc buffer (pH 3.0). After incubation at 37°C for 5 min, the color of the solution turned blue and its absorption at 652 nm was measured by UV-vis spectrophotometer.

#### Catalytic Reactions of Glucose

A series of concentrations of glucose (20 µl) and GOD (200 μl, 32.5 mM) were mixed in PBS and incubated at 37°C for 60 min, respectively. Then, 5 µl of Cu_2_O NPs (7 mM), 100 µl of TMB (20.8 mM), and 400 µl of acetate buffer (pH 3.0) were added to the above solution and incubated for 10 min for absorption measurement. For serum glucose detection, instead of glucose solution, a 20 µl serum sample was performed with the same experimental steps as mentioned above and the absorption values reflected the glucose concentration in serum samples due to the linear curve.

#### LDI Analysis of Glucose

One milliliter of glucose with concentrations ranging from 0.56 to 5.6 mM was mixed with an equal volume of cellobiose solution (2.9 mM), respectively. Then 500 nL of mixture solution and 500 nL of Cu_2_O NPs were dropped on the plate and analyzed by LDI-MS after drying. Cu_2_O NPs were dispersed in DI water at the concentration of 2.9 mM for use as a matrix. For LDI-MS analysis of glucose in serum, typically, the liquid of serum sample was mixed with equal volume cellobiose solution (2.9 mM) and spotted on the plate (1.5 µl) until dried in air at 25°C, followed by adding 1.5 µl of matrix and also dried in the same condition for LDI MS analysis.

### Results and Discussion

#### The Characterization of Cu_2_O NPs

**FIGURE 1:**
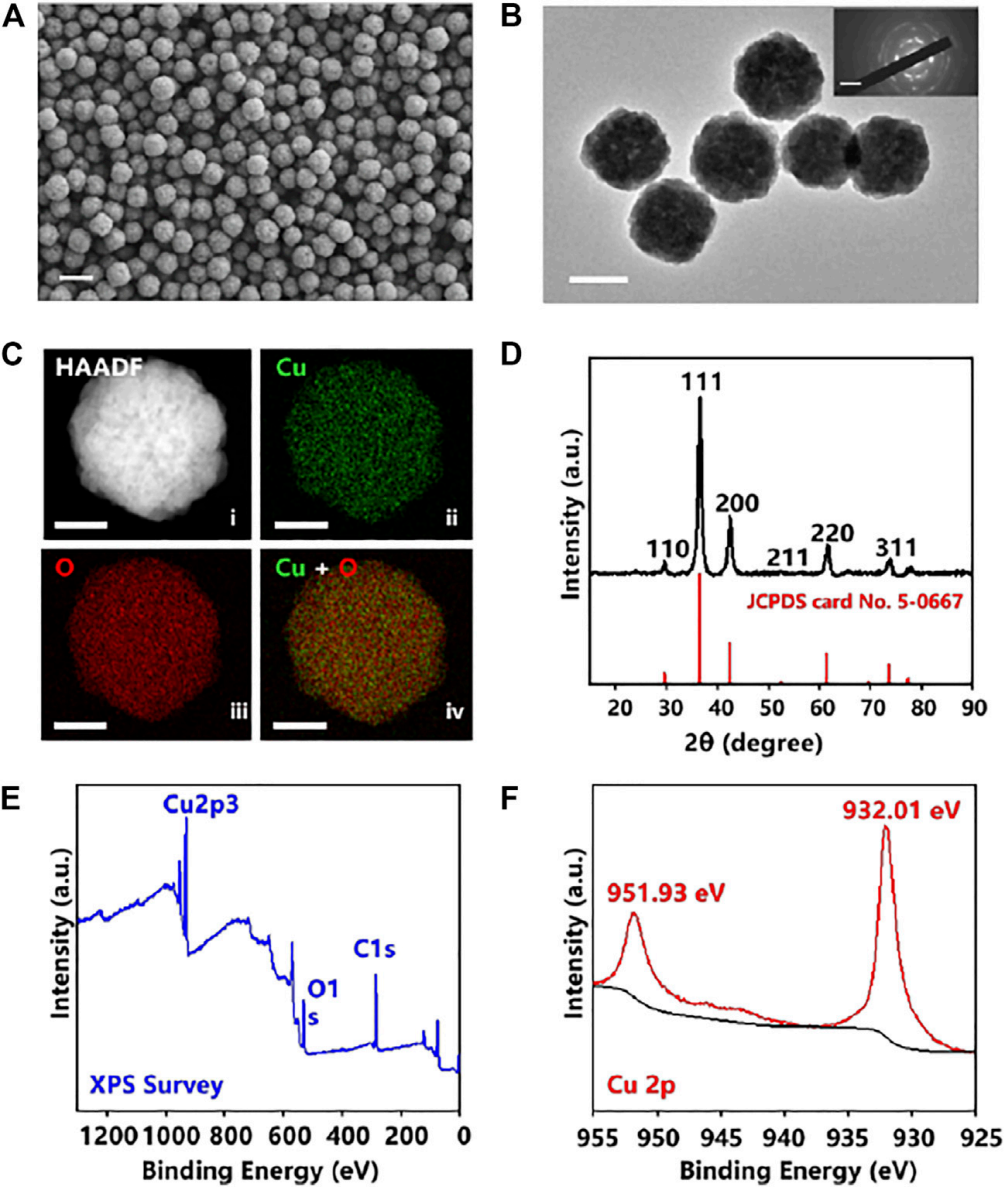
**(A)** SEM images of Cu_2_O NPs. The scale bar is 400 nm. **(B)** Transmission electron microscopy (TEM) image of Cu_2_O NPs (n ≥ 3 randomly selected) and selected area electron diffraction (SAED) pattern (inset) showing polycrystalline structure. The scale bar is 200 nm. **(C)** Elemental mapping of Cu_2_O NPs showing (ii) Cu in green, (iii) O in red, and (iv) overlapped Cu + O, with HAADF in (i), Scale bar is 100 nm. **(D)** XRD pattern of the Cu_2_O NPs. XPS spectra of the Cu_2_O NPs: **(E)** XPS Survey, and **(F)** Cu 2p.

The morphology of the prepared Cu_2_O NPs was characterized by SEM and TEM. As shown in Figures 1A,B, the Cu_2_O NPs exhibited a uniform spherical morphology with an average diameter of about 200 nm. Meanwhile, TEM images in Supplementary Figure S1 (ESI†) showed that the distribution of particles varied from 150 to 240 nm and was mainly concentrated on 200 nm. The selected area electron diffraction (SAED) pattern shown in Supplementary Figure S2 (ESI†) further confirmed the successful synthesis of pure Cu_2_O NPs (Hur et al., 2019). In Figure 1B, the surface of the Cu_2_O NPs was rough and its SAED (inset of Figure 1B) indicated that the Cu_2_O NPs have a polycrystalline structure (Chinnaiah et al., 2022). The elemental mapping of Cu_2_O NPs (Figure 1C) and the corresponding high-resolution transmission electron microscopy (HRTEM) image shown in Supplementary Figure S3 (ESI†) confirmed its high-quality polycrystalline nature (Huang and Luo, 2021). The phase structure and the purity of Cu_2_O NPs were analyzed using X-ray diffraction (XRD). As shown in Figure 1D, the diffraction peaks at a 2theta of 36.58, 42.40, 61.72, and 73.84 could be perfectly indexed to the lattices of Cu_2_O as (111), (200), (220), and 311) (JCPDS card No. 5-0667), respectively (Zhu et al., 2021). X-ray photoelectron spectroscopy (XPS) analysis was employed to further characterize the surface composition of the obtained Cu_2_O NPs. The XPS survey is shown in Figure 1E and Figure 1F for the high-resolution XPS spectrum of Cu 2p and the binding energy at 932.01 and 951.93 eV corresponds to Cu 2p_3/2_ and Cu 2p_1/2_, respectively, which are in good agreement with previous reports on Cu_2_O NPs (Pan et al., 2018; Zhang et al., 2020).

#### The Peroxidase-Like Activity of Cu_2_O NPs

**FIGURE 2:**
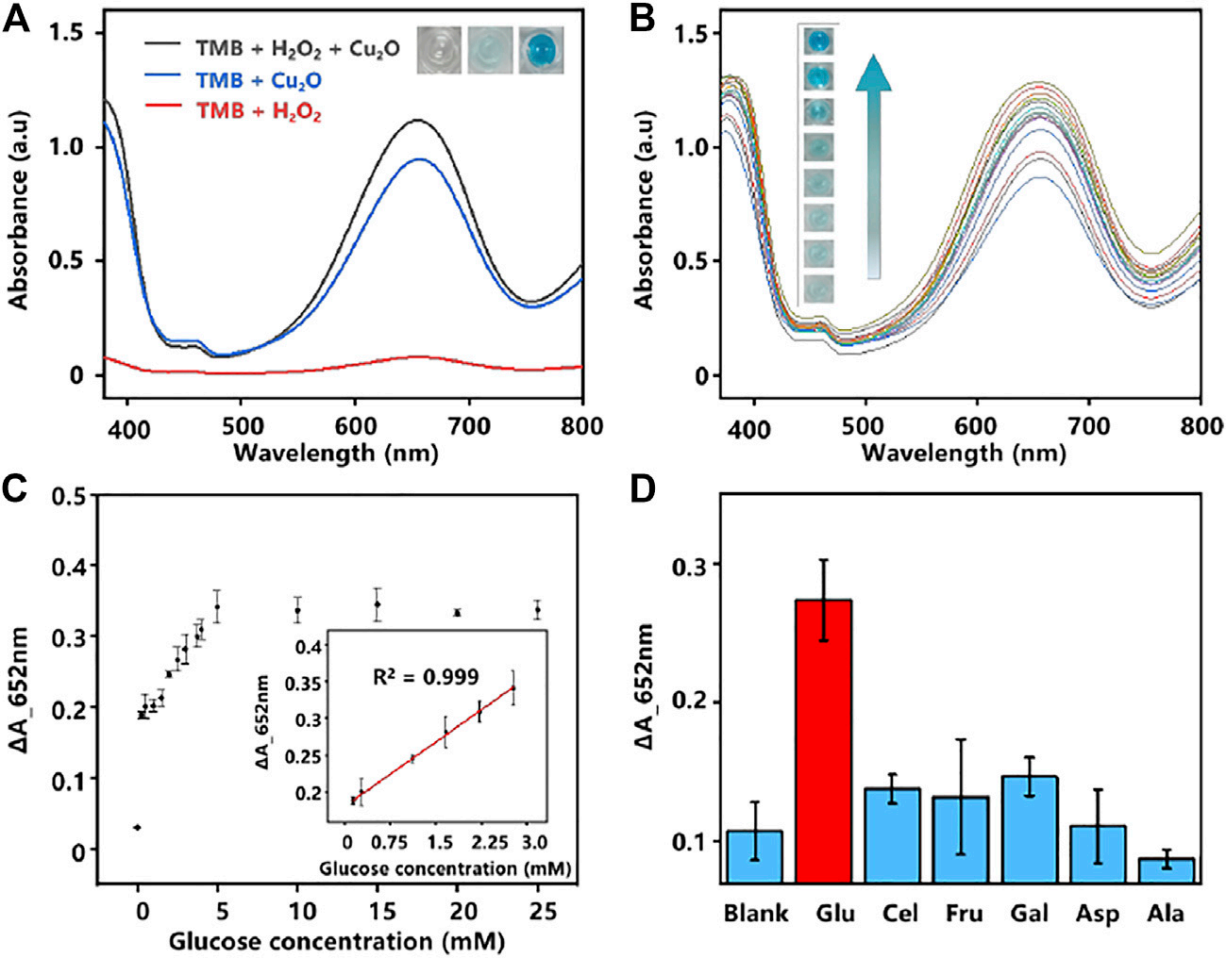
**(A)** UV-Vis absorption spectra of different systems. Inset images: color changes of the corresponding system. **(B)** UV-Vis absorption spectra of various concentrations of glucose were analyzed based on the GOD-Cu_2_O NPs-TMB system (0–10 mM, the interval of 1.25 mM from bottom to top). **(C)** The relationship plot and the linear curve (inset) between the concentration of glucose and the absorbance intensity at 652 nm. The error bars denote the SD of three measurements. **(D)** Selectivity analysis of the assay by monitoring the absorbance change of glucose and its analogs. The error bars denote the SD of five measurements.

To demonstrate the prepared Cu_2_O NPs possess the peroxidase-like catalytic activity as peroxidase mimics, the oxidation discoloration of TMB in the presence of H_2_O_2_ (TMB-H_2_O_2_ system) was selected (Liang et al., 2022). In the presence of the Cu_2_O NPs and H_2_O_2_, the peroxidase substrate TMB could be catalyzed to the oxidation state of TMB (ox TMB) by OH radicals produced from H_2_O_2_ and the solution will turn to blue (Zhang Z. et al., 2021). An electron paramagnetic resonance (EPR) analysis result shown in Supplementary Figure S4 (ESI†) further illustrated the existence of hydroxyl radicals in the system. As the results displayed in Figure 2A, an obvious absorption peak at 652 nm was observed when Cu_2_O NPs catalyzed TMB to ox TMB, while only a weak signal appeared in the absence of Cu_2_O NPs, indicating the good catalytic property of the Cu_2_O NPs.

#### Catalytic Reactions of Glucose by Cu_2_O NPs

Glucose is a source of energy for daily activities of the human body, while abnormal concentrations of glucose in the blood could lead to many endocrine metabolic diseases, such as diabetes (Senior, 2021). Given the high catalytic activity of the Cu_2_O NPs as peroxidase mimics for the TMB-H_2_O_2_ system, we constructed a glucose colorimetric biosensor based on the GOD-Cu_2_O NPs-TMB system. For glucose detection, the specific procedures are as follows. Firstly, GOD was used to oxidize glucose to gluconic acid and H_2_O_2_ in PBS (pH 7.4). Then, the *in situ* generated H_2_O_2_ was subsequently utilized to oxidize substrate TMB to chromogenic ox TMB with the Cu_2_O NPs in acetate buffer (pH 3.0). And the discoloration degree was recorded by microplate reader at the absorbance of 652 nm, which could reflect the concentrations of glucose. Using this method, a series of concentrations of glucose were detected and the results were displayed in Figure 2B. It can be observed that the color change was much more drastic as the concentration of glucose increased. Furthermore, a good linear relationship (*R* ^2^ = 0.999) between glucose concentration and the absorbance intensity of 652 nm was obtained (Figure 2C). The glucose colorimetric biosensor based on the GOD-Cu_2_O NPs-TMB system exhibited a broad linear range (0.28–2.8 mM) and the limit of detection (LOD) of the assay was 1.37 µM (S/N = 3), which is comparable or even superior to many recently reported nanocomposites in the literature (Zhao et al., 2015; Tan et al., 2017; Huang Y. et al., 2019; Kang et al., 2019; Vinothkumar et al., 2019). The detailed information is shown in Table 1.

**TABLE 1:** Comparison of linearity and LOD results for different materials-based glucose biosensors.

Material	LOD (µM)	Linear Range (mM)	Ref
Mn_2_O_3_ hollow NPs	2.46	0.01-0.1	Kang et al. (2019)
CeO_2_-TiO_2_	6.1	0.01-0.5	Zhao et al. (2015)
CePO_4_-CeO_2_	4.12	0-0.1	Vinothkumar et al. (2019)
CoO-OMC	68	0.1-5.0	Guo et al. (2019)
MnO_2_ nanowires	2	0.01-1	Han et al. (2017)
EPC	30	0.05-10	Ren et al. (2020)
MoO_3_/C	10	0.02-0.5, 0.5-6.0	Ren et al. (2019)
Cu_2_O NPs	1.37	0.28-2.8	This work

In the comparison with some other nanomaterial sensors for glucose detection (Han et al., 2017; Guo et al., 2019; Ren et al., 2019; Ren et al., 2020; Li et al., 2021; Liu et al., 2022), our proposed sensor shows a broad linear response in the range of 0.28–2.8 mM, which has wide prospects and great application values in the direct detection of glucose from human serum or other biological samples (Gluchowska et al., 2021). Currently, most glucose detection methods with low LOD can only realize standard samples analysis, while our work greatly achieved glucose detection from 31 serum samples. Besides, compared to most of the complicated nanoenzymes (Dong et al., 2016; Guo and Li, 2019; Zhang X. et al., 2021; Liu et al., 2021), an obvious advantage of Cu_2_O NPs as peroxidase mimic is their simple preparation and lower cost. Therefore, the prepared Cu_2_O NPs should be easily popularized and used in glucose detection.

In addition, selectivity was regarded as another major issue in analysis. We chose several carbohydrates (Cel, Fru, Gal, Asp, and Ala) to verify the selectivity of the assay for glucose detection (Dong et al., 2021). As shown in Figure 2D, different from glucose, no significant absorbance was observed from Cel, Fru, Gal, Asp, and Ala, demonstrating that the proposed system has excellent specificity for glucose analysis.

#### Cu_2_O NPs Based LDI MS Analysis of Small Molecules

**FIGURE 3:**
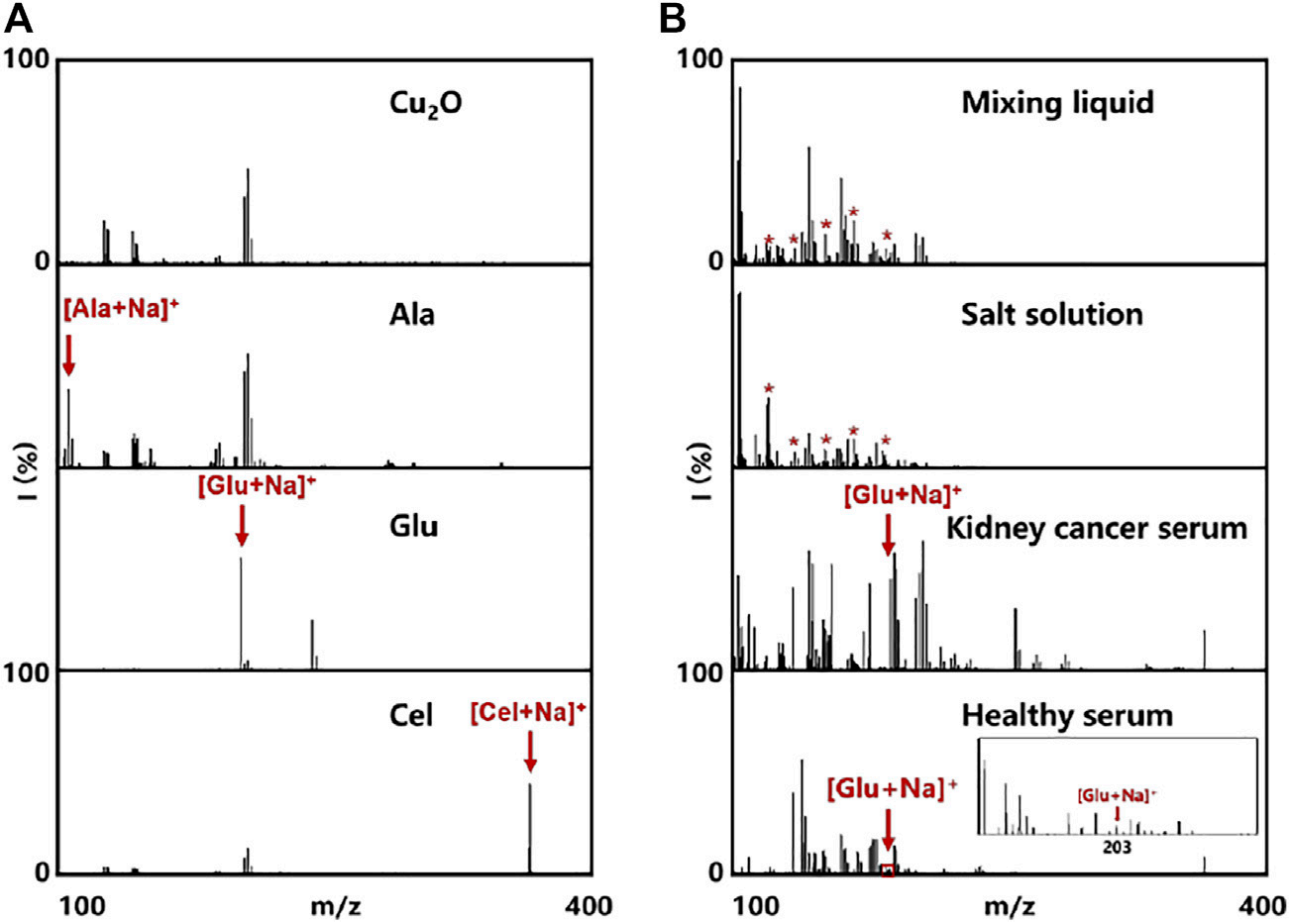
Cu_2_O NPs assisted LDI MS for analysis of **(A)** Cu_2_O NPs as control and some small metabolites (500 nL, 7 mM for each). **(B)** mixing liquid of alanine, leucine, aspartic acid, arginine, and glucose in water and salt solution, as well as metabolic fingerprinting of serum (500 nL) from healthy control and kidney cancer patient. The red asterisks represent alanine, leucine, aspartic acid, arginine, and glucose at the m/z peaks of 127, 154, 178, 199, and 203, respectively.

LDI MS presents high sensitivity, accuracy, resolution, and throughput in molecular analysis, especially for metabolites at the low molecular weight (Kulkarni et al., 2021). However, the efficiency of LDI MS relies on the matrix materials with designed molecular interfaces due to the size-exclusive effect and specific affinity (Ding et al., 2022). In this work, the optimized Cu_2_O NPs could not only be used as peroxidase mimic but also be employed as the matrix for LDI MS analysis. To testify the LDI MS performance based on Cu_2_O NPs, the typical small molecules were analyzed containing alanine, glucose, cellobiose (0.5 µL, 1 mg/ml, respectively). From the mass spectra in Figure 3A, we could clearly observe the peaks at m/z of 112, 203 and 365, corresponding to [Ala + Na]^+^, [Glu + Na]^+^ and [Cel + Na]^+^, respectively. Notably, the background signal from Cu_2_O NPs could not affect the detection of the above molecules.

Meanwhile, we also tested the Cu_2_O NPs based LDI MS for analysis of the mixing liquid in water and salt solution. As shown in Figure 3B, the peaks at m/z of 127, 154, 178, 199 and 203 were assigned to [Cho + Na]^+^, [Asp + Na]^+^, [DA + Na]^+^, [Vc + Na]^+^ and [Glu + Na]^+^, respectively, even in the interference of salt. Such results demonstrated the capability of multiple metabolites analysis and the good salts tolerance using Cu_2_O NP assisted LDI MS, which is hopefully applied in practical applications without pretreatment (Huang et al., 2017). To further evaluate the feasibility and practical applicability of glucose detection, serum samples from healthy adults and patients with kidney disease were tested directly by the Cu_2_O NPs assisted LDI MS. It could be apparently seen the peaks of glucose, indicating that our proposed system possesses the potential possibility of analyzing real blood samples.

#### Cu_2_O NPs Based LDI MS Quantitative Analysis of Glucose

**FIGURE 4:**
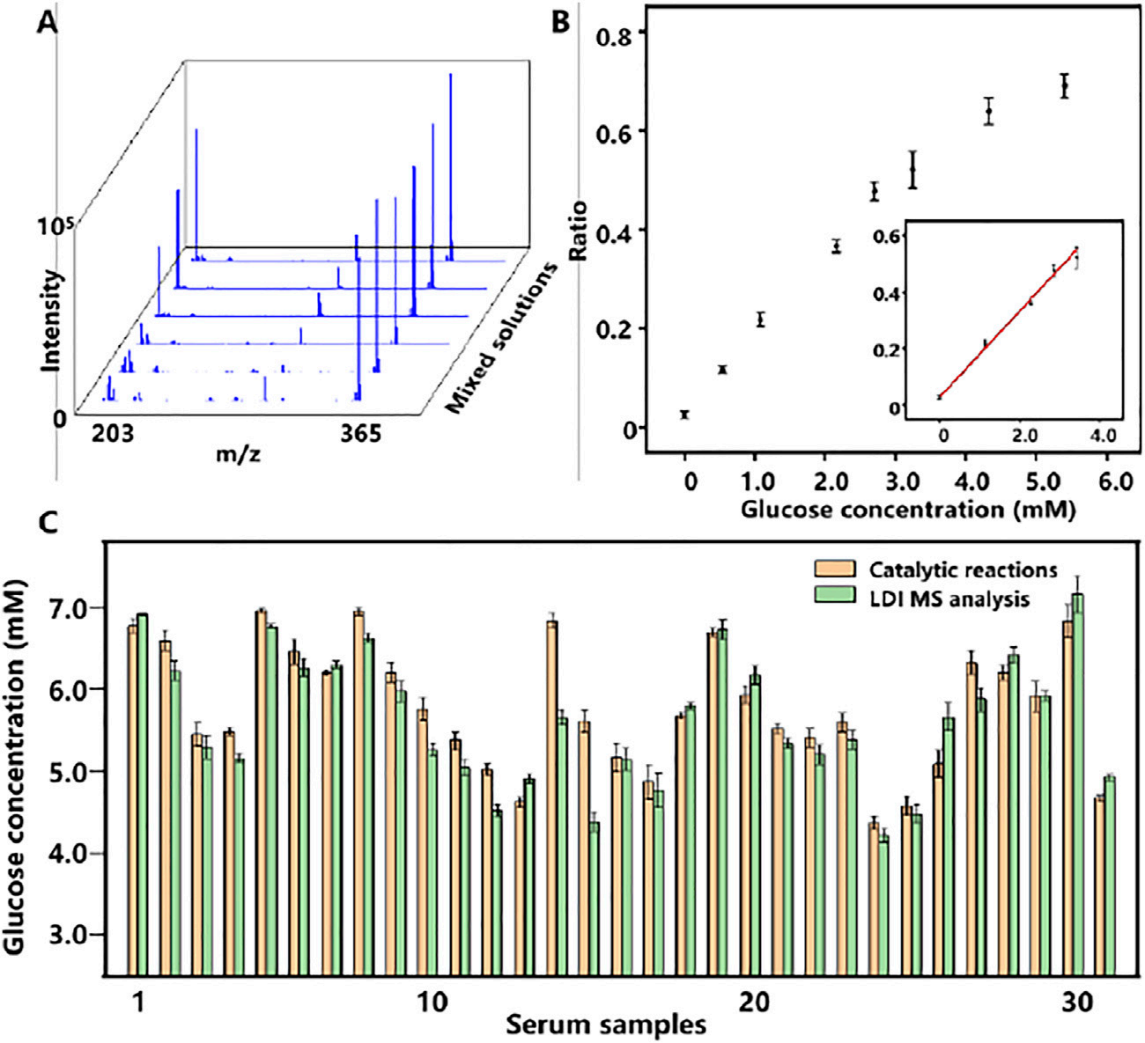
**(A)** LDI MS spectra of a gradient concentration of glucose and cellobiose (IS). **(B)** The relationship plot and the linear curve (inset) of the relative peak intensity of glucose (*I*_*203*_ */I*_*365*_) and the concentrations of glucose. The error bars denote the SD of three measurements. **(C)** Dual-mode glucose detection results of 31 serum samples. The error bars denote the SD of five measurements.

Encouraged by the good performance and salt tolerability of Cu_2_O NPs assisted LDI MS analysis of metabolites, we further detected glucose from serum samples with this method. For accurate quantitative analysis of glucose by LDI MS, cellobiose with a similar ionization efficiency to glucose was selected as internal standard (IS). Firstly, different concentrations of glucose ranging from 0.56 to 5.6 mM were mixed with cellobiose (2.9 mM) and analyzed by Cu_2_O NP based LDI MS. The results in Figure 4A displayed the peaks of glucose at m/z of 203 and cellobiose at m/z of 365. Moreover, the relative intensity of glucose (*I*_*203*_ */I*_*365*_) was increased as the concentration of glucose increased and a good linear relationship (*R* ^2^ = 0.997) between the *I*_*203*_ */I*_*365*_ and the concentration of glucose from 0 to 3.36 mM was obtained in Figure 4B.

#### Dual-Mode Quantitative Analysis of Glucose in Serum

Considering that glucose level in serum is associated with various diseases in the clinic, quantitative evaluation of glucose from serum is meaningful. To demonstrate the feasibility of dual-mode quantitative of glucose from serum, 31 serum samples were prepared and analyzed by both colorimetric and LDI MS assay based on Cu_2_O NPs shown in Figure 4C. The glucose concentrations of serum samples were distributed from 4.12 to 7.26 mM, basically in line with the range of normal blood glucose values in healthy people (3.61–7.77 mM). Notably, the difference between the two methods was less than 0.5 mM with a correlation coefficient of 0.902 in Supplementary Figure S5 (ESI†).

## Conclusion

In summary, we developed a dual-mode Cu_2_O NPs based biosensor for glucose analysis by integrating colorimetric assay and LDI MS. The Cu_2_O NPs were simply prepared and low-cost, which presented excellent peroxidase-like activity and assisted LDI MS analysis of metabolites. The Cu_2_O NP based dual-mode biosensor was successfully applied in visual and quantitative analysis of glucose from serum, demonstrating the potential values in clinical diseases diagnostics and monitoring.

## Data Availability

The original contributions presented in the study are included in the article/Supplementary Material, further inquiries can be directed to the corresponding authors.
