# Supplementary material for: A Copper-Based Biosensor for Dual-Mode Glucose Detection
Source: Front Chem. 2022 Apr 4;10:861353. doi: 10.3389/fchem.2022.861353 (PMC9014126; doi:10.3389/fchem.2022.861353)
Supplement: Supplementary file 1 [file DataSheet1.docx]

A copper-based biosensor for dual-mode glucose detection

This part included:

Figure S1-S5

# Supplementary Figures


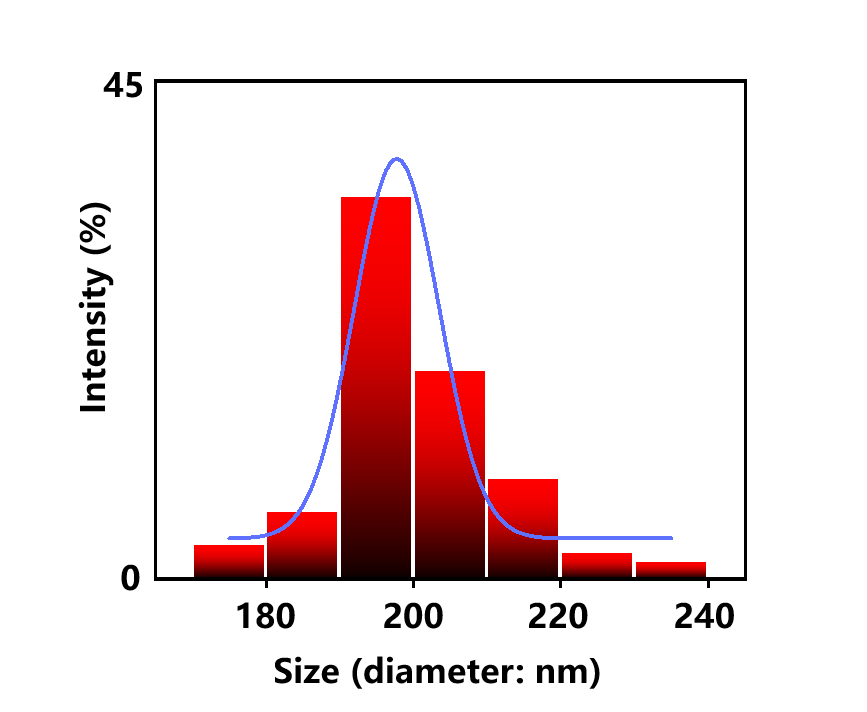


**Supplementary Figure 1.** Cu_2_O NPs size distribution. The size distribution was dependent on 3 independent measurements.

**
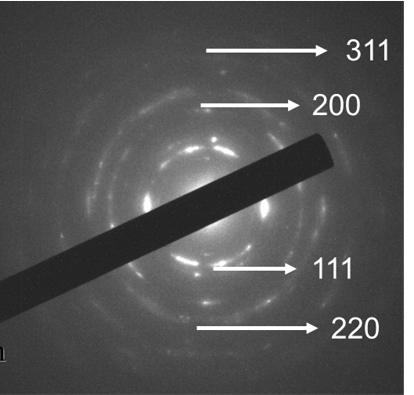
**

**Supplementary Figure 2.** Selected area electron diffraction (SAED) pattern of Cu_2_O. The results were

shown with the SAED pattern along [111], [200], [220], and [311] directions.


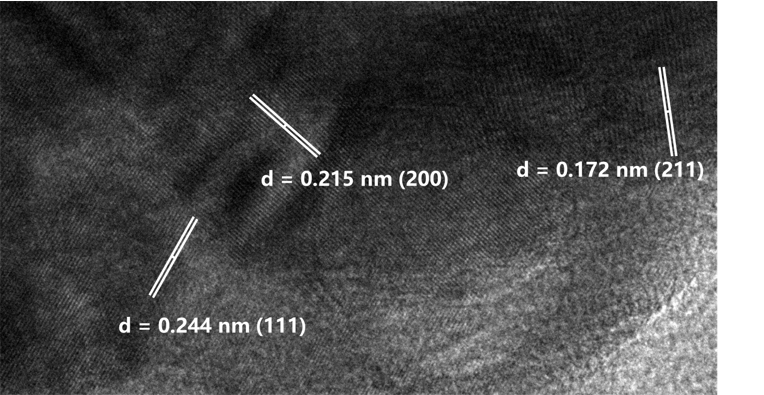


**Supplementary Figure 3.** High-resolution transmission electron microscopy (HRTEM) image

showed the Cu_2_O NPs crystal lattice. The typical interplanar spacing of 0.172 nm, 0.215 nm, and 0.244

nm for these particles was observed, corresponding with the SAED pattern of [211], [200], and [111],

respectively.


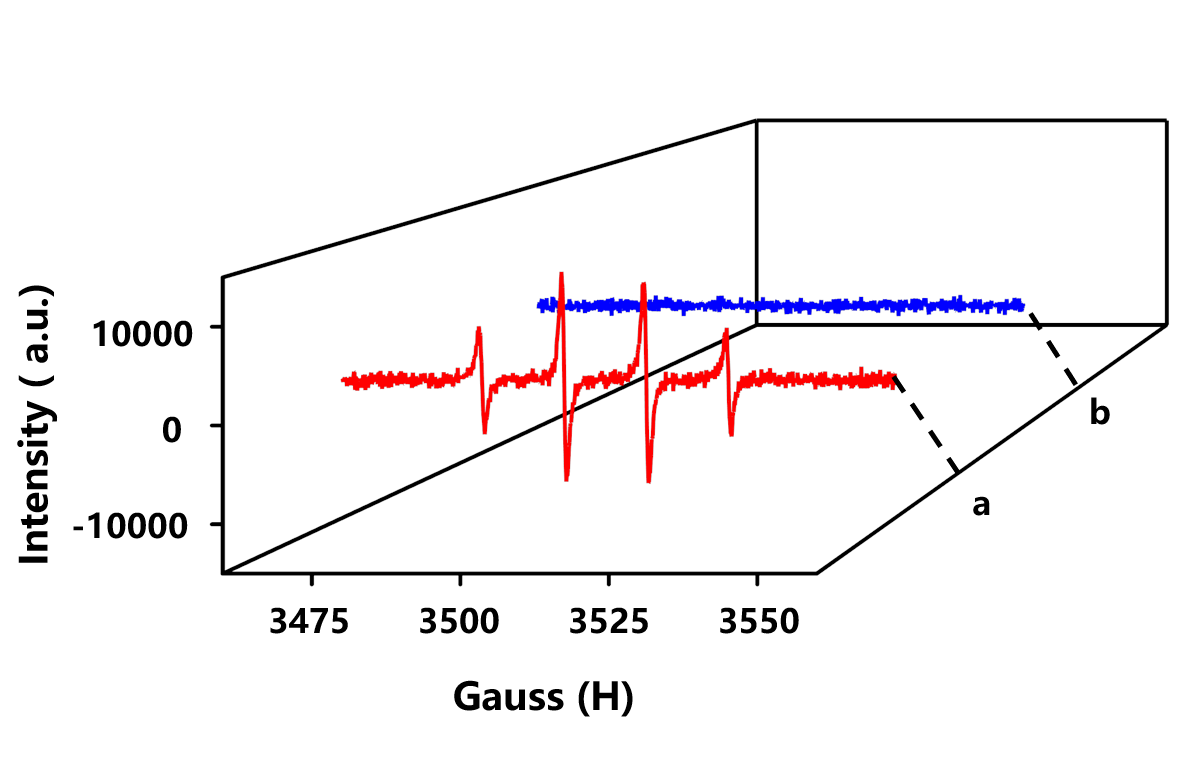


**Supplementary Figure 4.** EPR spectra of 0.1M PBS containing 0.1M H_2_O_2_ in the presence with (a) 10 µL 1mg/mL Cu_2_O nanoparticles and (b) 10 µL DI water, respectively.


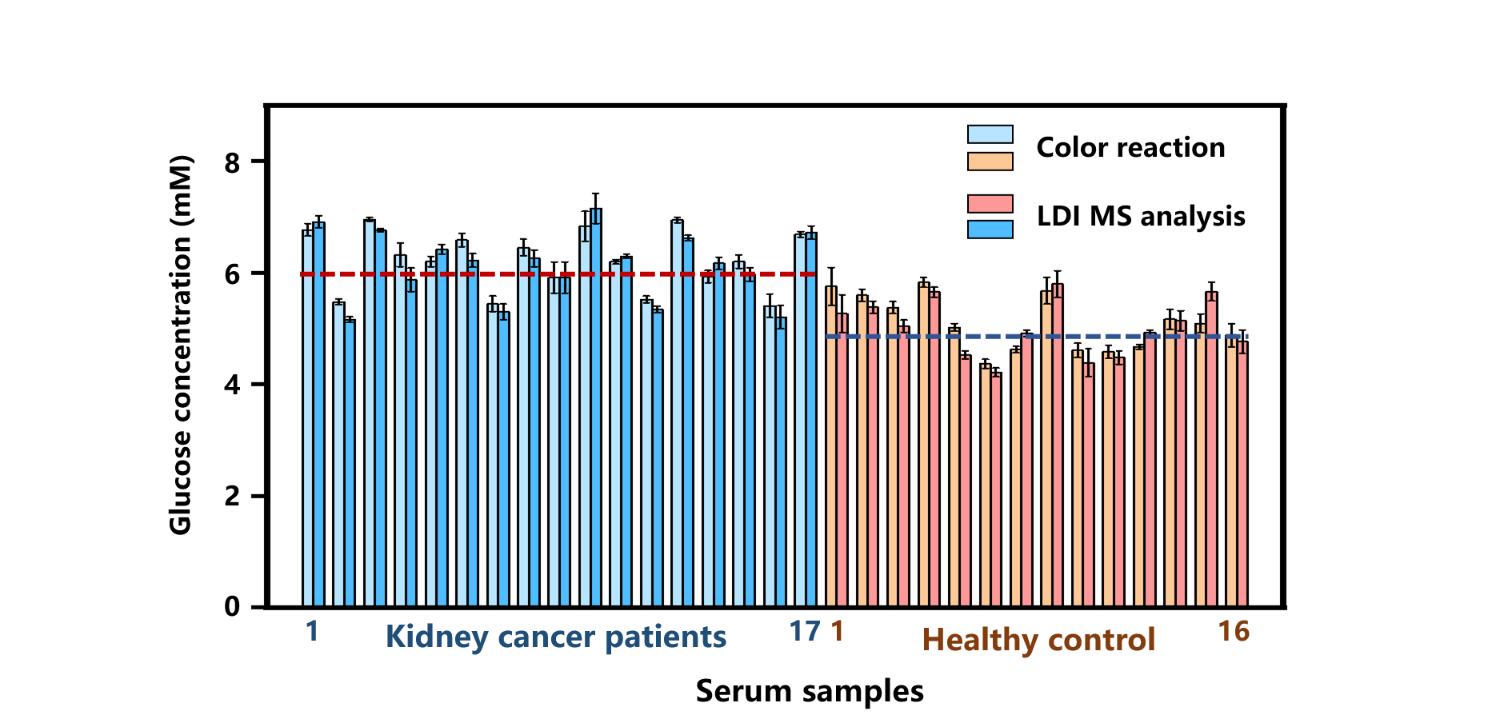


**Supplementary Figure 5.** Dual-mode serum glucose detection results of 16 healthy controls and 17

kidney cancer patients.
